# 11791 Gray matter volume differences in bilingual compared to monolingual children

**DOI:** 10.1017/cts.2021.457

**Published:** 2021-03-30

**Authors:** Alison K. Schug, Edith Brignoni-Perez, Nasheed Jamal, Guinevere F. Eden

**Affiliations:** Center for the Study of Learning, Department of Pediatrics, Georgetown University Medical Center

## Abstract

ABSTRACT IMPACT: This study examines gray matter volume differences resulting from the bilingual experience in children and adults allowing us to better understand the brains of over half of the world’s population that speaks more than one language. OBJECTIVES/GOALS: Literature is mixed regarding a bilingual advantage in executive control (EC). While it has been shown that young adult bilinguals have greater gray matter volume (GMV) than monolinguals in EC regions, there is behavioral evidence that suggests such difference would be more pronounced in children. METHODS/STUDY POPULATION: Using SPM12 to test this hypothesis, we used a whole-brain t-test to compare GMV in 35 English-speaking monolingual and 20 Spanish-English early (learned both languages before 6 years old) bilingual children. Next, we submitted both groups of children to an ANOVA with 42 English speaking monolingual and 26 Spanish-English bilingual adults to test for an interaction of Language Experience by Age Group at the level of the whole brain. RESULTS/ANTICIPATED RESULTS: e between-group comparison of bilingual and monolingual children, revealed more GMV in bilingual compared to monolingual children in regions associated with EC (right middle and inferior frontal gyri, superior parietal lobule, and precuneus). Our second analysis, an ANOVA comparing bilingual and monolingual children and adults, revealed an interaction in which bilingual>monolingual GMV in children was greater than any bilingual>monolingual GMV (or bilingual=monolingual GMV) in the adult groups in the right superior parietal lobule (BA1). No regions indicated that bilingual>monolingual GMV was more pronounced in adults. DISCUSSION/SIGNIFICANCE OF FINDINGS: These results provide further evidence for GMV differences in early bilinguals in regions associated with EC and indicate that more GMV differences exist between bilingual and monolingual children than adults.

